# Plasma expression of HIF‐1α as novel biomarker for the diagnosis of obstructive sleep apnea‐hypopnea syndrome

**DOI:** 10.1002/jcla.23545

**Published:** 2020-09-08

**Authors:** Caidong Liu, Haoyu Wang, Chenbin Zhu, Shukui Wang

**Affiliations:** ^1^ Department of Laboratory Medicine Nanjing First Hospital Nanjing Medical University Nanjing China; ^2^ Department of Biochemistry and Molecular Biology Nanjing Medical University Nanjing China

**Keywords:** HIF‐1α, obstructive sleep apnea‐hypopnea syndrome, polysomnography

## Abstract

**Background:**

Obstructive sleep apnea‐hypopnea syndrome (OSAHS) is a common breathing disorder during sleep with potential lethality and multi‐complications. Polysomnography (PSG) is now the golden standard for the diagnosis obstructive sleep apnea‐hypopnea syndrome. However, PSG is expensive and time‐consuming. Therefore, it is important to find inexpensive and convenient biomarkers for the diagnosis of OSAHS.

**Objective:**

The present study aimed to explore the potential diagnostic value of HIF‐1α for OSAHS and its clinical significance.

**Methods:**

This study consisted of 368 patients admitted to the sleep laboratory. The patients were classified according to their apnea‐hypopnea index (AHI) scores as OSA negative (AHI < 5), mild‐moderate (AHI:5‐30), and severe OSA (AHI > 30), and severe OSA treated with continuous positive airway pressure (CPAP). qRT‐PCR was used to detect mRNA levels in the plasma; Pearson's correlation analysis was performed to analyze the correlation of HIF‐1α mRNA level and the clinicopathological factors of OSAHS; ROC curve was constructed to evaluate the diagnostic value of HIF‐1α mRNA.

**Results:**

HIF‐1α mRNA was significantly up‐regulated in the plasma of OSAHS patients, especially patients with severe OSAHS. HIF‐1α mRNA was positively correlated with the AHI and ODI but negatively correlated with the mean oxygen saturation in patients with OSAHS. Results of ROC curve showed that HIF‐1α is a sensitive biomarker for the diagnosis of OSAHS, especially severe OSAHS.

**Conclusions:**

HIF‐1α mRNA might be used as s a convenient and inexpensive method for triaging OSAHS patients PSG assessment in the hospital and evaluate the curative effect.

## Graphical abstract

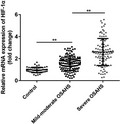

## 1 INTRODUCTION

Obstructive sleep apnea‐hypopnea syndrome (OSAHS) is a common sleep apnea characterized by the snore and repeated episodes of complete or partial collapse of the upper airway during sleep.^1^, ^2^ The incidence of OSAHS increased significantly in recent years, especially among the elderly.^3^, ^4^, ^5^ Based on previous reports, long‐term OSAHS condition can increase the risk for the incidence of metabolic diseases and cardiovascular diseases (CVD) such as heart failure, hypertension, and arrhythmias.^6^, ^7^, ^8^

In current clinical application, polysomnography (PSG) is the golden standard for the diagnosis of OSAHS.^11^ However, PSG is expensive and time‐consuming. Therefore, it is of vital importance to find easy and inexpensive methods for the early diagnosis of OSAHS. Many factors have been reported to contribute to the pathogenesis of OSAHS, and inflammation induced by the repeated hypoxia‐reoxygenation has been considered as one of the key factors during the development of OSAHS.^9^, ^10^ Hypoxia‐inducible factor‐1α (HIF‐1α), the subunit of HIF‐1, plays very important roles in the hypoxia related signaling pathway by regulating different cellular and molecular events.^12^ It has been reported that HIF‐1 was up‐regulated in patients with OSAHS^13^, ^14^; however, it remains unclear whether OSAHS may serve as biomarker for the diagnosis and treatment of the disease. Therefore, in this study, we focused on the potential diagnostic values of HIF‐1 in OSAHS.

## 2 MATERIALS AND METHODS

### 2.1 Study subjects and demographic characteristics

This study included data of 368 individuals with snore problem from sleep center in Nanjing First Hospital, Nanjing Medical University (Jiangsu, China), between April 2016 and October 2018. Patients were classified according to their apnea‐hypopnea index (AHI) scores as OSA negative (AHI < 5), mild‐moderate (AHI:5‐30), and severe OSA (AHI > 30). Moreover, 96 patients with severe OSA and treated with continuous positive airway pressure (CPAP) were also included. The exclusion criteria were as follows: (a) psychiatric or neuropathic causes of sleep disorder; (b) diagnosed with metabolic disorders, endocrine, autoimmune disorders; (c) respiratory diseases including asthma and acute respiratory tract infection; (d) diagnosed with liver or kidney disease; (e) hematologic disorders including anemia and leukemia; and (f) age < 18 years. The demographic characteristics such as age, gender, and body mass index (BMI) were collected. BMI was calculated by dividing weight by height squared. Cardiovascular diseases (CVD) were patients who suffered from heart failure, arrhythmia, or coronary artery disease. Diabetes mellitus (DM), hypertension (HT), and smoking history was defined as the risk factor of CVD. This study has been approved by the ethical committee of Nanjing First Hospital, and every patient has signed the inform consent form.

### 2.2 Sleep recording

Sleep recording was performed by using overnight polysomnography (PSG), comprising continuous recording electroencephalography (EEG), electrooculography (EOG), submental and bilateral anterior tibial electromyography (EMG), electrocardiography (ECG), nasal airflow, and pulse oximetry. A physical examination including weight and height was recorded before PSG. Sleep stages and respiratory events during sleep were scored based on the guideline of American Academy of Sleep Medicine (AASM).^15^ Apnea was defined as a ≥90 decrease of airflow for more than 10 seconds; hypopnea was defined as a ≥50% decrease of airflow amplitude compared to the baseline, or a ≥30% decrease in the airflow for 10 seconds associated with a decrease in oxygen saturation for ≥3% or an EEG arousal. Apnea‐hypopnea index (AHI) was the number of apnea or hypopnea per hour during sleep. The oxygen desaturation index (ODI) was defined as the total number of measurements of oxyhemoglobin desaturation of ≥4% within ≥10 seconds to <3 minutes from the baseline, divided by the total sleep time.

### 2.3 RNA extraction and qRT‐PCR

Total RNAs were extracted from the plasma by TRIzol reagent (Invitrogen) following the manufacturer's instructions. Next, the purity and quantity of the total RNA were detected with DeNovix DS‐11 and a Spectrophotometer (DeNovix). RNA was reverse‐transcribed to cDNA from 1 µg of total RNA by using AMV reverse transcriptase (Takara) and a RT primer according to the manufacturer's recommendation. The reaction conditions were as follows: 16°C for 30 minutes, 42°C for 30 minutes, and 85°C for 5 minutes. Real‐time PCR (RT‐PCR) was carried out by using a Taqman PCR kit on an Applied Biosystems 7300 sequence detection system (Applied Biosystems), with U6 as the internal control. The reactions were performed in a 96‐well plate at 95°C for 10 minutes, followed by 40 cycles of 95°C for 10 seconds and 60°C for 1 minutes.

### 2.4 Statistical analysis

The software of SPSS 20.0 was used for statistical analyses, and all of counting data were expressed in the form of mean ± standard deviation(x ± SD). One‐way ANOVA was used for analyses of interclass variance, and LSD *t* test was performed in the multiple comparison. Receiver operating characteristic (ROC) analysis was performed, and areas under curve were calculated to evaluate the diagnostic value of HIF‐1 mRNA. All tests of statistical analysis are two sides, with *P* < .05 taken to statistical significance.

## 3 RESULTS

### 3.1 Clinical characteristics and sleep parameters in patients

A total of 368 patients were included in the study, among which 48 individuals were diagnosed as simple snore, 122 patients were mild‐moderate OSAHS, 102 patients were severe OSAHS, and 96 patients with severe OSA and treated with continuous positive airway pressure (CPAP). Table 1 and Table 3 displayed the demographic data and PSG parameters of the participators in the groups. The BMI increased gradually in parallel with the severity of OSAHS (*P* < .01). The results of the polysomnographic study showed no significance on sleep stage I of the patients (*P* = .8353), but there have been significant differences in sleep stage II time/TST and sleep stage III time/TST and sleep efficiency among different groups. Moreover, the AHI and ODI increased while the mean and minimum oxygen saturation decreased with the severity of OSAHS, which was statistical differences between severe OSAHS and other groups. SE showed no significant differences among the groups (*P* = .0747).

**Table 1:** Demographic and polysomnographic findings of the study group

	Control (n = 48)	Mild‐Moderate (n = 112)	Severe (n = 102)	*P*‐value
Age (years)	51.2 ± 5.3	51.9 ± 5.6	50.7 ± 4.9	.2511
Gender (Male/Female)	23/25	62/50	54/48	.6881
BMI (kg/m^2^)	26.8 ± 3.2	31.5 ± 4.1	33.6 ± 3.6	<.001
Sleep parameters				
Stage 1	4.7 ± 1.9	4.6 ± 2.2	4.5 ± 1.7	.8353
Stage 2	52.1 ± 5.3	54.2 ± 6.7	67.5 ± 7.2	<.001
Stage 3	23.7 ± 3.5	25.2 ± 4.3	17.6 ± 4.1	<.001
REM	16.5 ± 2.4	15.2 ± 3.6	14.3 ± 3.9	.0019
SE	81.5 ± 9.2	83.7 ± 10.6	85.4 ± 9.3	.0747
AHI	2.6 ± 1.2	17.4 ± 2.9	60.5 ± 10.9	<.001
Mean O_2_ sat	107.2 ± 20.5	93.7 ± 9.5	85.4 ± 7.2	<.001
ODI	2.4 ± 0.9	12.6 ± 3.4	46.2 ± 6.8	<.001

Abbreviations: AHI, Apnea‐hypopnea index; ODI, oxygen desaturation ındex; REM, Rapid eye movement; sat, saturation; SE, Sleep efficiency.

### 3.2 HIF‐1α was up‐regulated in the plasma of patients with severe OSAHS

**FIGURE 1:**
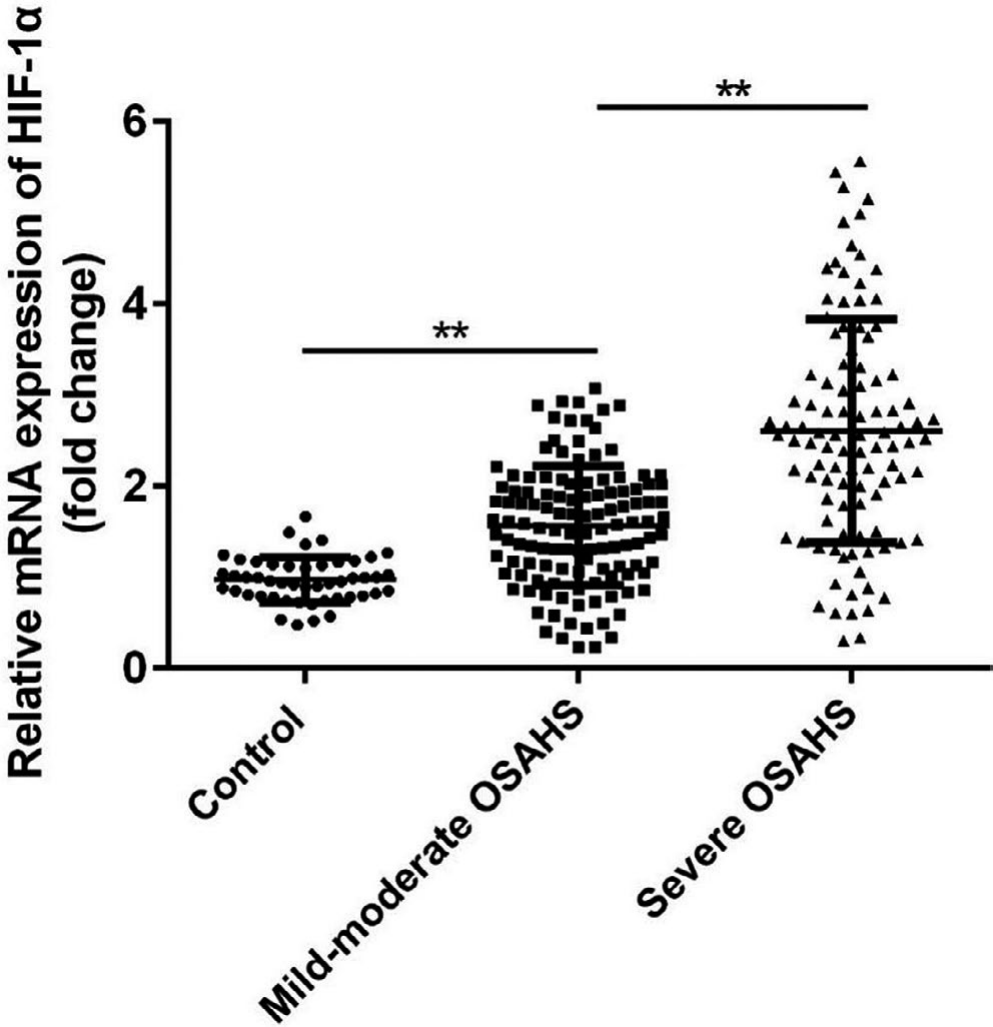
HIF‐1α mRNA expression levels in the plasma OSAHS patients. **P < .01

First, the mRNA expression of HIF‐1αin plasma samples of the patients and healthy controls in each group was measured by qRT‐PCR methods. As shown in Figure 1, the levels of HIF‐1α were significantly increased in the plasma samples of both mild and severe OSAHS patients compared with the controls (Figure 1, *P* < .01); moreover, the level of HIF‐1α was significantly increased in severe OSAHS group compared with the mild‐moderate group (Figure 1, *P* < .01).

### 3.3 HIF‐1α may serve as potential diagnostic marker for OSAHS

**FIGURE 2:**
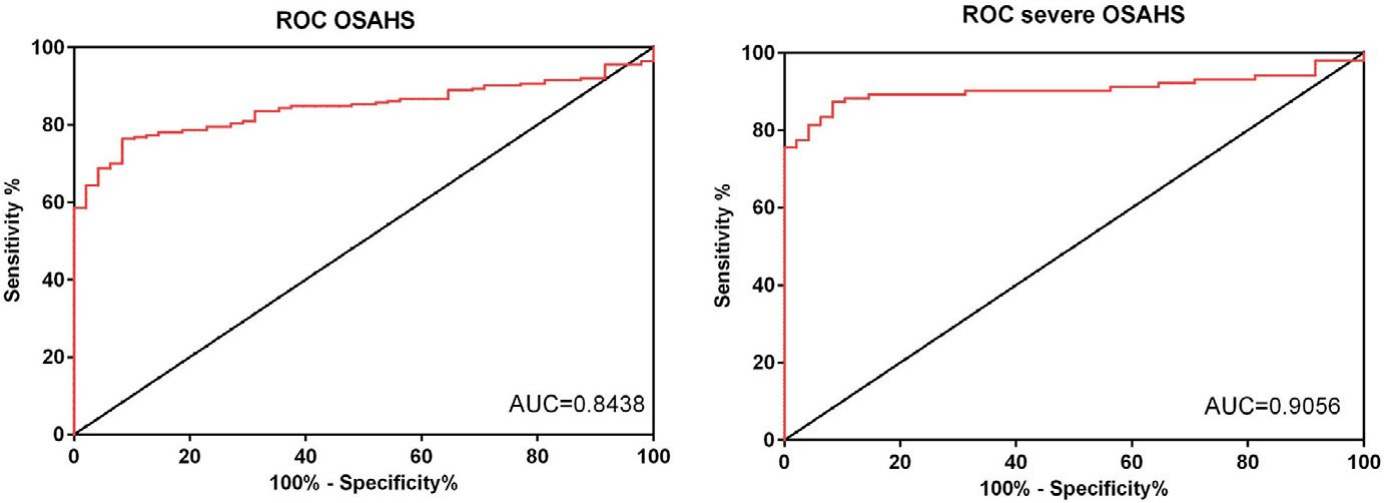
HIF‐1α may serve as potential diagnostic marker for OSAHS

Moreover, we performed ROC curve analysis to identify whether HIF‐1α mRNA can serve as a biomarker in OSAHS. The analysis revealed that the area under the ROC curve (AUC) for distinguishing the OSAHS patients and controls was 0.8438 (Figure 2, 95% confidence interval (CI) 0.7985‐0.8890), and the AUC for distinguishing the severe OSAHS and controls was 0.9056 (Figure 2, 95% CI, 0.8549‐0.9564).

### 3.4 HIF‐1α is correlated with clinicopathological factors in patients with OSAHS

**FIGURE 3:**
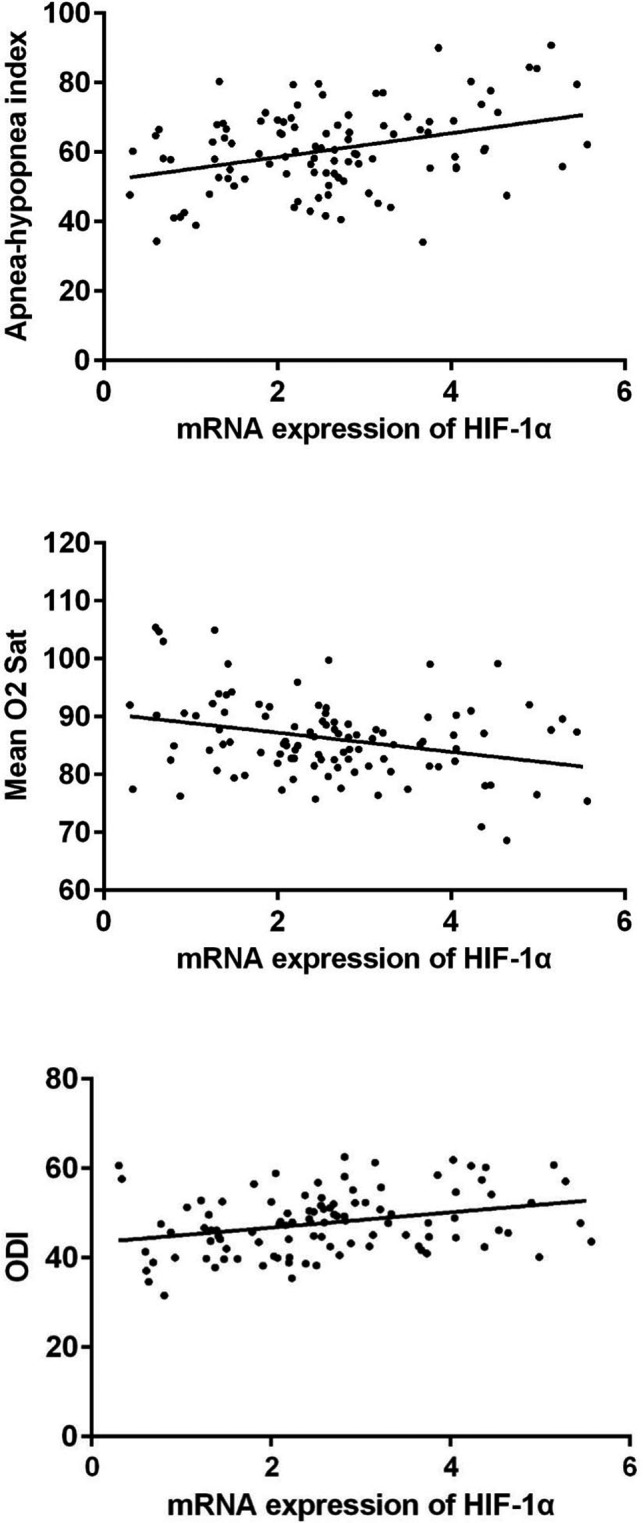
Correlation between HIF‐1α mRNA and polysomnographic parameters

Next, we also investigated the correlations between polysomnographic results and laboratory parameters. Pearson's correlation analysis showed a significant correlation between HIF‐1α mRNA level and polysomnographic parameters. HIF‐1α mRNA was positively correlated with the AHI and ODI but negatively correlated with the mean oxygen saturation (*P* < .001; Figure 3 and Table 2).

**Table 2:** Correlation between HIF‐1α mRNA and polysomnographic parameters

	*r*	*P*
AHI	.3523	.0003
Mean O_2_ sat	−.2963	.0025
ODI	.3051	.0018

Abbreviations: AHI, Apnea‐hypopnea index; ODI, oxygen desaturation index; sat, saturation.

### 3.5 HIF‐1α was decreased after CPAP treatment in patients with OSAHS

**FIGURE 4:**
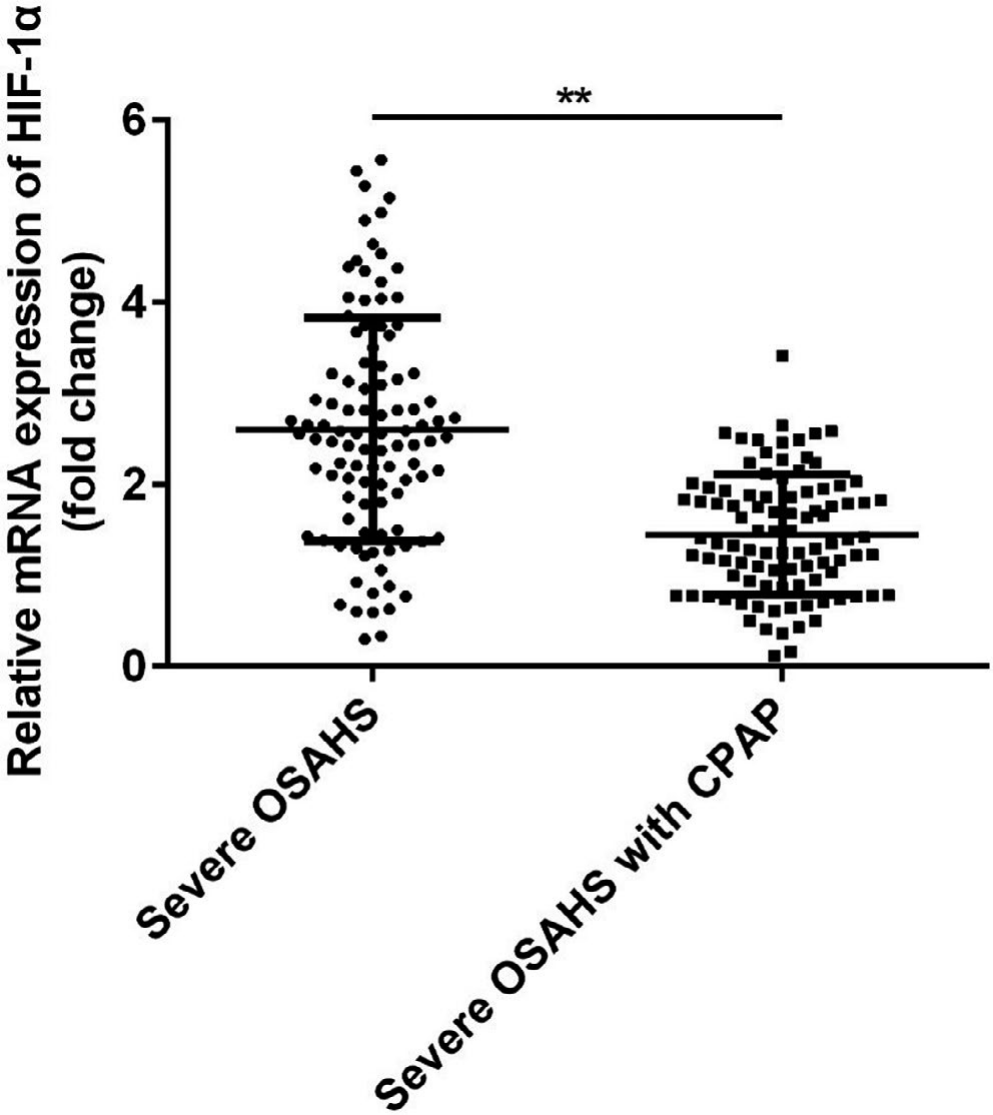
HIF‐1α was decreased after CPAP treatment in patients with OSAHS. **P < .01

Finally, the plasma levels of HIF‐1α in severe OSAHS patients with or without CPAP treatment were compared. The clinical characteristics of patients in these two groups were shown in Table 3. We found that most of the sleeping parameters were changed by CPAP treatment. Moreover, as shown in Figure 4, severe OSAHS patients who had got CPAP treatment showed a lower plasma HIF‐1 mRNA level compared with the severe OSAHS patients, suggesting that may serve as potential biomarker for evaluating the therapeutic efficacy for OSAHS.

**Table 3:** Demographic and polysomnographic findings of the CAPA and non‐CAPA groups

	Severe (n = 102)	Severe + CAPA (n = 96)	*P*‐value
Age (years)	50.7 ± 4.9	51.3 ± 3.8	.3389
Gender (Male/female)	54/48	55/41	.5385
BMI (kg/m^2^)	33.6 ± 3.6	33.9 ± 2.8	.5153
NASH (Yes/No)	73/29	68/28	.9091
Sleep parameters			
Stage 1	4.5 ± 1.7	4.6 ± 2.3	.7272
Stage 2	67.5 ± 7.2	55.3 ± 6.1	<.001
Stage 3	17.6 ± 4.1	24.5 ± 3.2	<.001
REM	14.3 ± 3.9	15.7 ± 1.4	.0014
SE	85.4 ± 9.3	83.4 ± 7.8	.1038
AHI	60.5 ± 10.9	16.2 ± 3.7	<.001
Mean O_2_ sat	85.4 ± 7.2	94.1 ± 8.6	<.001
ODI	46.2 ± 6.8	13.1 ± 4.2	<.001

Abbreviations: AHI, Apnea‐hypopnea index; NASH, nonalcoholic steatohepatitis; ODI, oxygen desaturation ındex; REM, Rapid eye movement; sat, saturation; SE, Sleep efficiency.

## 4 DISCUSSION

Obstructive sleep apnea‐hypopnea syndrome is a multisystem respiratory disease which is potentially lethal and occurred with many complications including cardiovascular diseases, metabolism dysfunction, pulmonary diseases, and stroke.^16^, ^17^, ^18^, ^19^ The underlying mechanism of OSAHS remains unclear, but chronic inflammation induced by the hypopnea has been considered as an important reason during the occurrence and development of OSAHS.^20^ Here in the present studies, we found that HIF‐1α, an inflammatory regulator, is up‐regulated in OSAHS, and the level of HIF‐1α was associated with clinicopathological factors in patients with OSAHS. The current data proposed the possibility of HIF‐1α as a potential biomarker for the early diagnosis of OSAHS before PSG assessment or evaluating the therapeutic efficacy of the treatment to OSAHS.

Intermittent hypoxia is one of the typical features of OSAHS.^21^, ^22^, ^23^ Hypoxia induces the production of reactive oxygen species, resulting in inflammation and endothelial dysfunction.^24^ So biomarkers of oxidative stress and inflammation may be applied for evaluating OSAHS and other related disease. Stanke et al reported that leukotriene E4 excretion increased in obstructive sleep apnea patients.^25^ Goldbart et al found that children with obstructive sleep apnea syndrome exhaled more inflammatory mediators.^26^ Here in the present studies, we found HIF‐1α mRNA was significantly up‐regulated in the plasma of OSAHS patients, especially severe OSAHS patients compared to the control group. HIF‐1 is a key mediator of hypoxic signaling and was a urinary biomarker of kidney disease.^27^ Turcotte et al reported that HIF‐1α up‐regulated during hypoxia in renal cell carcinoma.^28^ However, HIF‐1α protein showed no significant correlations with AHI.^29^ In the present work, we found a significant decrease of HIF‐1α mRNA in the plasma of severe OSAHS patients who had been treated with CPAP, the gold standard therapy that delivers pressurized air into the upper airway to relieve obstruction during sleep,^30^ indicating that HIF‐1α mRNA may serve as an indicator for the therapeutic efficacy. Then, we performed Pearson's correlation analysis to confirm the correlation between the mRNA expression of HIF‐1α and polysomnographic parameters. An increased HIF‐1α mRNA may also be proposed as a simple and diagnostic tool for pre‐screening the severity of OSAHS patients. The ROC curve showed a good diagnostic value of HIF‐1α mRNA for OSAHS, especially severe OSAHS.

The current work has limitations. First, the number of the patients in the present studies is still limited, especially for the control group; Second, more biomarkers still need to be found to increase the accuracy of the diagnose. Third, a multivariable model between HIF‐1α levels and cofounders such as AHI, ODI, TST90 and clinical comorbidities should also be investigated to further elucidate the role of HIF‐1α in OSAHS.

In conclusion, our results indicated that HIF‐1α mRNA was correlated with the severity of OSAHS, and HIF‐1α mRNA can be used as a convenient and inexpensive method for the diagnosis of OSAHS patients PSG assessment in the hospital and evaluate the curative effect. Based on current findings, the diagnostic roles of HIF‐1α mRNA for patients with other respiratory diseases (eg, COPD) + OSAHS could also be investigated in future works.

## CONFLICT OF INTEREST

None.
